# Effect of Age on the Biomechanical Properties of Porcine LCL

**DOI:** 10.3390/bioengineering12010005

**Published:** 2024-12-24

**Authors:** Narendra Singh, Jovan Trajkovski, Jose Felix Rodriguez Matas, Robert Kunc

**Affiliations:** 1 Chair of Modelling in Engineering Sciences and Medicine, Faculty of Mechanical Engineering, University of Ljubljana, Aškerčeva c. 6, 1000 Ljubljana, Slovenia; jovan.trajkovski@fs.uni-lj.si; 2 Department of Chemistry, Materials and Chemical Engineering “Giulio Natta”, Politecnico di Milano, Piazza Leonardo da Vinci, 32, 20133 Milano, Italy; josefelix.rodriguezmatas@polimi.it

**Keywords:** porcine ligament, tension test, age-related changes, Lateral Collateral Ligament, biomechanics

## Abstract

The Lateral Collateral Ligament (LCL), one of the four major ligaments in the knee joint, resides on the outer aspect of the knee. It forms a vital connection between the femur and the fibula. The LCL’s primary role is to provide stability against Varus forces, safeguarding the knee from undue rotation and tibial displacement. Uniaxial mechanical testing was conducted on the dog bone (DB) samples in this study. The porcine of different ages, from 3 months to 48 months (4 years) old, were used to analyse the effect of age. A constant head speed of 200 mm/s was applied throughout the tests to mimic strain–stress and damage responses at an initial strain rate of 13.3/s. The mechanical properties of LCL were evaluated, with a specific focus on the effect of age. The LMM (Linear Mixed Model) analysis revealed a marginally significant positive slope for Young’s modulus (*p* = 0.0512) and a significant intercept (*p* = 0.0016); for Maximum Stress, a negative slope (*p* = 0.0346) and significant intercept (*p* < 0.0001); while Maximum Stretch showed a significant negative slope (*p* = 0.0007) and intercept (*p* < 0.0001), indicating the muscle reduces compliance and load-bearing capacity with age.

## 1. Introduction

Tendons and ligaments are intricate connective soft tissues with an organized arrangement, serve the crucial roles of upholding joint stability and facilitating joint mobility. As individuals advance in age, these specific tissues undergo structural and compositional modifications, which consequently exert substantial influence on their biological characteristics, regenerative potential, and biomechanical efficacy [1]. The ultimate strength of the ligament tends to diminish as age increases [2]. Conversely, stiffness displays augmentation with the progression of age [3]. The process of aging induces alterations in the diameter of collagen fibres and triggers the relaxation of collagen fibrils that are typically crimped in nature [4,5]. Both the structural attributes of bone–ligament–bone (BLB) complexes (frequently employed for tensile evaluations of ligaments) and the inherent material properties of ligamentous substances experience a decline due to the aging process [2,6,7,8,9]. This phenomenon was observed in studies involving both animal models [2] and human subjects [6,7,8,9].

**Figure 1:**
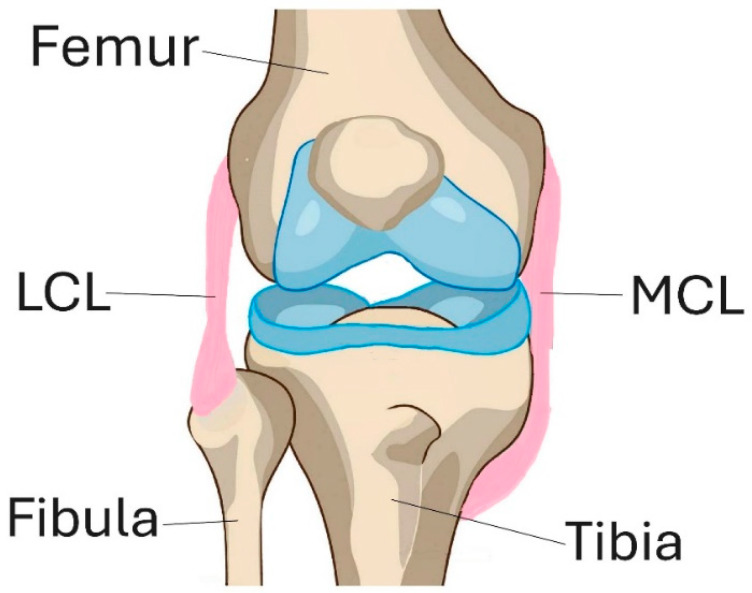
LCL and MCL ligaments in knee.

The lateral collateral ligament (LCL) is one of the four major ligaments of the knee joint. It is located on the outer side of the knee and connects the femur to the fibula, as shown in Figure 1. The LCL primarily acts as the primary Varus stabilizer of the knee joint. It prevents excessive lateral movement (Varus stress) and posterolateral rotation of the tibia relative to the femur. When the cruciate ligaments (ACL and PCL) are torn, the LCL serves as a secondary stabilizer for anterior and posterior tibial translation. It is particularly significant for Varus rotation within the range of 0–30° of knee flexion. While the ligament’s indispensability is widely recognized, the intricate interplay between aging and the LCL remains a relatively unexplored area. Limited research in this field obscures our ability to comprehend how age-related physiological changes may manifest within the LCL.

The biomechanical properties and morphometric characteristics of the porcine Lateral Collateral Ligament (LCL) do indeed change with age. A study carried out by Cone [10] investigated the changes in the length and cross-sectional area of four ligaments and tendons, including the LCL, in the tibiofemoral joint of female Yorkshire pigs throughout growth. Interestingly, the LCL showed allometric growth, indicating a preference for greater changes in length rather than cross-sectional area. Regarding the mechanical behaviour of the LCL ligament, studies in the literature are rather scarce. In Bonner et al. [11], the porcine stifle joint, which closely resembles the human knee joint in terms of morphology, size, structure, material properties, and physiological loading is studied. The work involved healthy female large-white pigs, aged between 9 and 12 months, to explore the mechanical properties of the LCL. Strain rates ranging from 0.01 to 100/s were considered, indicating that the material properties of the LCL are sensitive to strain rates up to a limit of approximately 1/s. However, more specific studies on the biomechanical properties of the porcine LCL influenced by age are needed for a comprehensive understanding. To the best of the authors knowledge, no study was found on the effect of age on the biomechanical properties of a porcine LCL.

In this study, we conducted uniaxial mechanical testing on dog-bone (DB) samples of LCL (Pure ligament testing). The goal was to evaluate the mechanical properties, specifically focusing on the effect of age on mechanical properties of LCL at an initial strain rate of 13.3/s. In this study, an initial strain rate of 13.3 s^−1^ was chosen to replicate the dynamic loading conditions that soft tissues usually experience in applications such as sports activities or collisions. These scenarios often involve intermediate high strain rates, ranging from 10 s^−1^ to 100 s^−1^. In real-world scenarios, engineering structures and materials often experience rapid loading, as seen in traffic accidents or collisions [12]. By selecting this strain rate, we can accurately analyse the mechanical behaviour of soft tissues under such conditions, providing valuable insights into their response to dynamic forces. We aimed to understand how the LCL responds to loading and how its behaviour varies across different ages. This research contributes to our understanding of ligament biomechanics and provides valuable insights for clinical applications and treatment strategies. In this study, we focus on three important markers to analyse the effect of age and they are Youngs modulus, maximum stress and maximum stretch. These parameters are pivotal as they provide essential insights into the structural behaviour of materials under different conditions, particularly regarding age-related changes. Firstly, the Youngs modulus serves as a foundational measure, reflecting the material’s stiffness at the onset of loading. It indicates the material’s ability to resist deformation initially, offering a baseline for understanding how it responds to stress over time. Secondly, the maximum stress is a critical factor in assessing material strength. It signifies the highest level of stress that the material can endure before failure, providing valuable information on its overall resilience and durability. Lastly, the maximum stretch indicates the extent to which the material can deform before failure. Understanding this parameter is crucial for predicting the material’s performance and longevity, especially in applications where deformation tolerance is a key consideration.

## 2. Material and Methods

### 2.1. Sample Preparation

The porcine stifle joint ligaments were selected due to their anatomical and mechanical similarity to human knee ligaments, including comparable morphology, size, structure, and loading characteristics [13]. Thirteen hind limbs from healthy female Landrace pigs, aged 3 to 48 months, were obtained from a local slaughterhouse [14]. This specific age range and controlled selection minimized variations in material properties related to sex, breed, and health status [15]

The experimental protocol commenced with the collection of freshly dissected Lateral Collateral Ligament (LCL) samples within 2–3 h post-mortem. These specimens were immediately transported to the laboratory and stored at −20 °C to preserve their integrity because at this temperature it is possible to maintain the physical and mechanical properties of the specimens [16,17]. Each ligament was sealed in a plastic bag containing 0.9% saline solution, which helped maintain the tissue’s hydration. The chosen preservation method was informed by previous research [18,19,20] to ensure that the mechanical and physical properties of the ligaments were retained.

**Figure 2:**
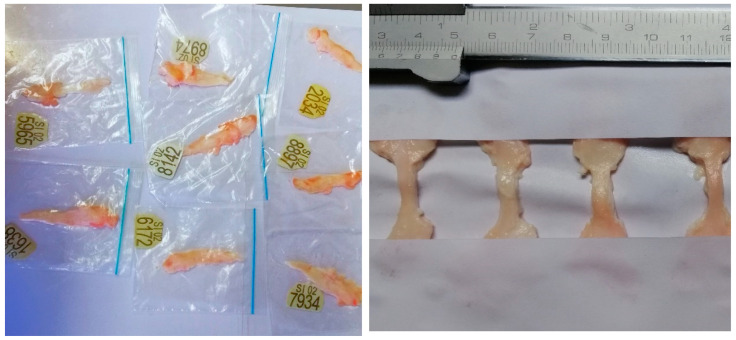
LCL sample preparation.

Approximately an hour after initial freezing, the samples were removed for precise cutting. The frozen state of the ligaments enhanced the accuracy of cuts, as the rigidity allowed for consistent shaping and sizing, which is essential for reliable mechanical testing. Medical scissors and a knife were used to carefully clean any uneven surfaces, including the removal of the upper envelope of the soft tissue to expose the true ligament underneath. After cleaning and cutting, the initial length was consistently set to 15 mm, while the width varied depending on the age of the specimen ranging from 5 to 15 mm This step provided a smooth surface ideal for marking the dots required for strain rate calculations. Additionally, tissue paper was used to gently tap the ligament surface to make it moisture free, as moisture can distort the shape of the marker complicating the tracking. Figure 2 shows LCL ligaments stored in plastic bags (left) and after precise cutting (right).

Before testing, the samples underwent a carefully controlled thawing process. The frozen ligaments were allowed to thaw slowly at room temperature while sealed in plastic bags filled with 0.9% saline solution.

### 2.2. Testing Procedure

**Figure 3:**
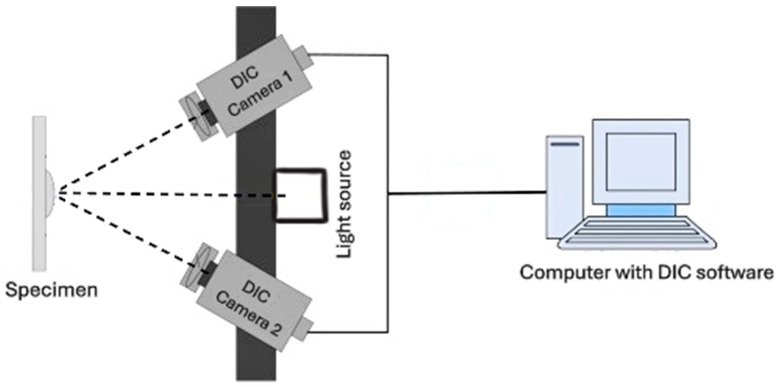
Schematic of uniaxial testing along with DIC camera setup.

Uniaxial testing provides a fundamental assessment of the mechanical properties of tissues when subjected to uniaxial loading conditions. In this study, a multiaxial testing machine, manufactured by Step Labs, Italy [21], was utilized to conduct these evaluations. The machine can achieve speeds of up to 1 m/s. To secure the specimens during testing, Inox clamps were employed, ensuring a firm grip on the tissue. A schematic of uniaxial testing along with DIC camera setup can be seen in Figure 3. A preliminary preload of 20 N was applied to the samples to eliminate any slack and provide a consistent starting reference.

**Figure 4:**
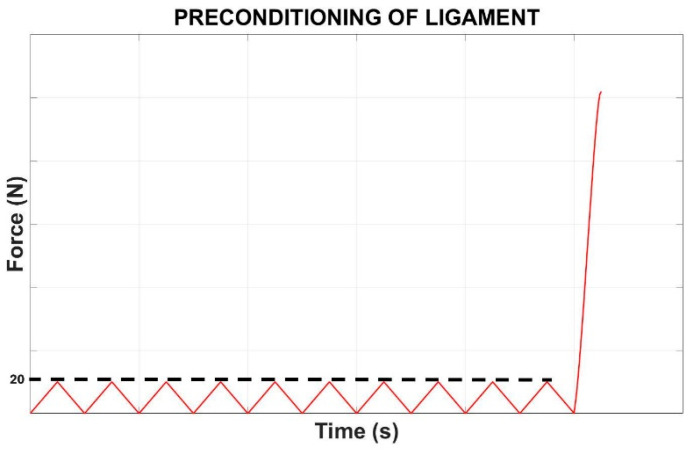
Preconditioning of ligament.

Stainless steel clamps were used to hold the sample in place, and a 20 N preload was applied to the tissue from a slack starting position. The testing protocol began with a series of 10 preconditioning cycles as shown in Figure 4. This preconditioning phase involved cyclically loading the ligament from 0 to 20 N at a controlled velocity of 100 mm/s. The purpose of preconditioning is to stabilize the mechanical response of biological tissues by reducing the effects of stress relaxation, viscoelasticity, and plastic deformation before actual testing. This step is crucial for standardizing the mechanical state of the specimen and enhancing the reliability of the data collected in subsequent tests.

**Figure 5:**
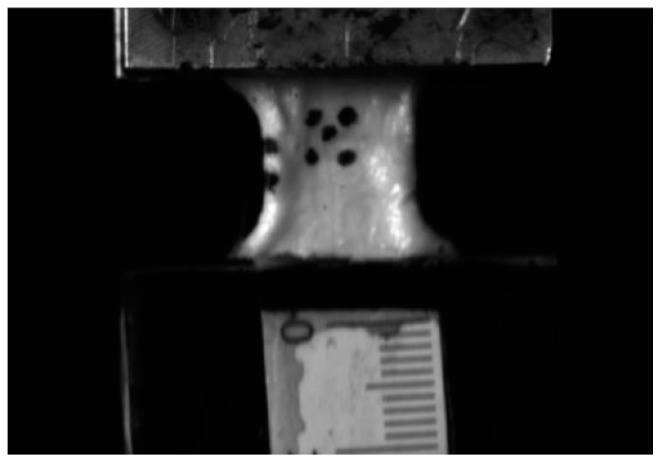
DIC system LCL uniaxial testing.

After preconditioning, the initial measurements of the ligament, including length, width, and thickness, were obtained under a nominal preload of 20 N. These measurements were performed using high-resolution images acquired with Digital Image Correlation (DIC) cameras from Dantec Dynamics [22]. These cameras enabled precise mapping of the ligament’s dimensions, providing a critical baseline for subsequent analysis as shown in Figure 5.

**Figure 6:**
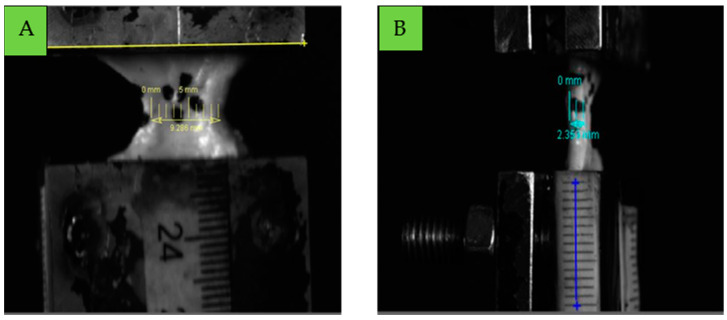
Width (**A**) and thickness (**B**) measurement in tracker software.

The cross-sectional area was measured using Tracker software version 6.2.0 [23] an open-source tool specifically designed for motion tracking, to analyse the recorded footage. First, a known reference length was measured within the software to calibrate the scale for accuracy. For the ligament width measurement, we used scale as well as the known width of an inox clamp (35 mm) Figure 6A, and to measure the thickness a paper scale attached to the clamp was used as shown in Figure 6B. The width and thickness sections of the ligament were recorded. In this study, the cross-sectional area was calculated by multiplying the measured width by thickness, assuming a rectangular cross-section. This simplification was chosen to ensure consistent and reproducible measurements across all specimens, enabling a standardized approach for calculating mechanical properties.

Once the dimensional measurements were completed, the 20 N preload was released, and a tensile load was applied at a controlled head speed of 200 mm/s until ligament failure occurred. Throughout this phase, the DIC cameras were utilized exclusively for capturing the deformation markers on the specimen, the advanced optical capabilities of the DIC cameras provided real-time data on changes in length, width, and thickness of the LCL dog bone samples during the tensile loading. We used 50 mm focal length for the cameras and videos were captured at 250 fps. Every video has a resolution of 1588 × 720 pixels. This configuration enabled simultaneous monitoring of the ligament’s longitudinal deformation on one side and thickness changes on the opposite side. However, we did not employ DIC software for stress–strain calculations due to the uneven surface of the LCL ligament, which could compromise the accuracy of the measurements.

**Figure 7:**
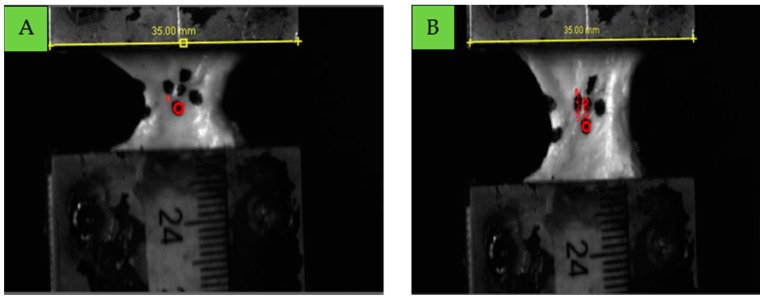
Strain measurement in tracker software, undeformed (**A**) and deformed (**B**) state.

By tracking the markers in Tracker software, we were able to accurately calculate the strain experienced by the ligament during the testing process. Figure 7A illustrates the initial position of the markers on the ligament in its undeformed state, establishing a baseline for displacement measurements. In contrast, Figure 7B depicts the ligament in its deformed state, demonstrating the displacement of the markers under applied load. This comparison between the initial and deformed states enables accurate calculation of strain in the ligament tissue. As shown in Figure 6, five markers were placed on the ligament surface, with three vertically aligned markers tracked for strain rate calculations. The average strain rate was determined by calculating the strain rates between these markers, using the central marker as a common reference point across all measurements. All experiments were performed within 4–5 min of removing the sample from the saline solution to maintain full hydration. Experimental procedures were conducted at a controlled room temperature to ensure consistent environmental conditions and eliminate temperature-related variations affecting the ligaments’ mechanical properties.

### 2.3. Statistical Analysis

Based on load elongation data (*F-*∆l), initial cross-sectional area ($A_{0}$) and initial length ($l_{0}$), First Piola stress–stretch curves (*P-λ*) were computed.

(1) $$P=\frac{F}{A_{0}}$$

(2) $$\lambda =1+\varepsilon =1+\frac{∆l}{l_{0}}$$

Once we obtained the stress vs. stretch curves, we focused on understanding the effect of age on three mechanical markers of ligaments, namely: (i) Youngs modulus, (ii) maximum stress, (iii) maximum stretch.

To better understand the relationships between age and the mechanical properties of LCL (Young’s modulus, maximum stress, and maximum stretch), we performed a Linear mixed model (LMM) analysis using MATLAB (R2021a—academic use). In this analysis, LMMs were used to study how Young’s modulus, maximum stress, and maximum stretch change with age, with output including slopes, intercepts and the corresponding *p*-values, which shows whether the correlations are statistically significant. A *p*-value less than 0.05 was considered significant. In this study, *p*-values were computed using the linear mixed-effects model (LMM) approach. This statistical method evaluates the relationship between mechanical properties (e.g., Young’s modulus, Maximum Stress, and Maximum Stretch) and age by estimating the coefficients of the independent variables. The *p*-values were obtained through the Wald *t*-test, which assesses the null hypothesis that the coefficients (e.g., the effect of age) are equal to zero. This test determines the statistical significance of the observed relationships. The LMM approach provides a robust framework for analysing trends while accounting for variability within the data.

## 3. Results

**Figure 8:**
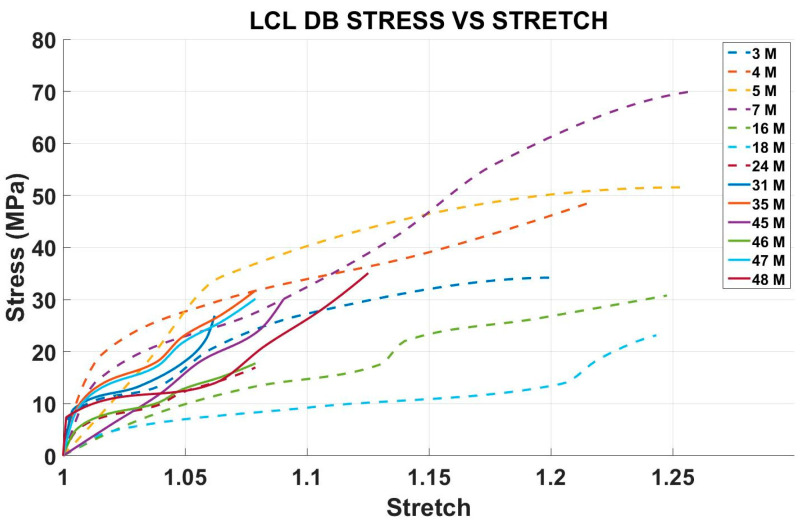
Stress vs. stretch ratio diagram of different age porcine LCLs.

The experimental stress versus stretch curves for 13 fresh Lateral Collateral Ligament (LCL) samples are shown in Figure 7. Encompassing specimens from a diverse age range of 3 to 48 months. Each sample underwent a standardized testing protocol to ensure consistency and reproducibility across all tests. Relatively younger samples under the age of 25 months are shown with dotted lines while samples above the age of 25 months are shown with regular lines. From Figure 8, it can be seen that, in general, younger ligaments exhibit higher stretch values and flexibility.

**Figure 9:**
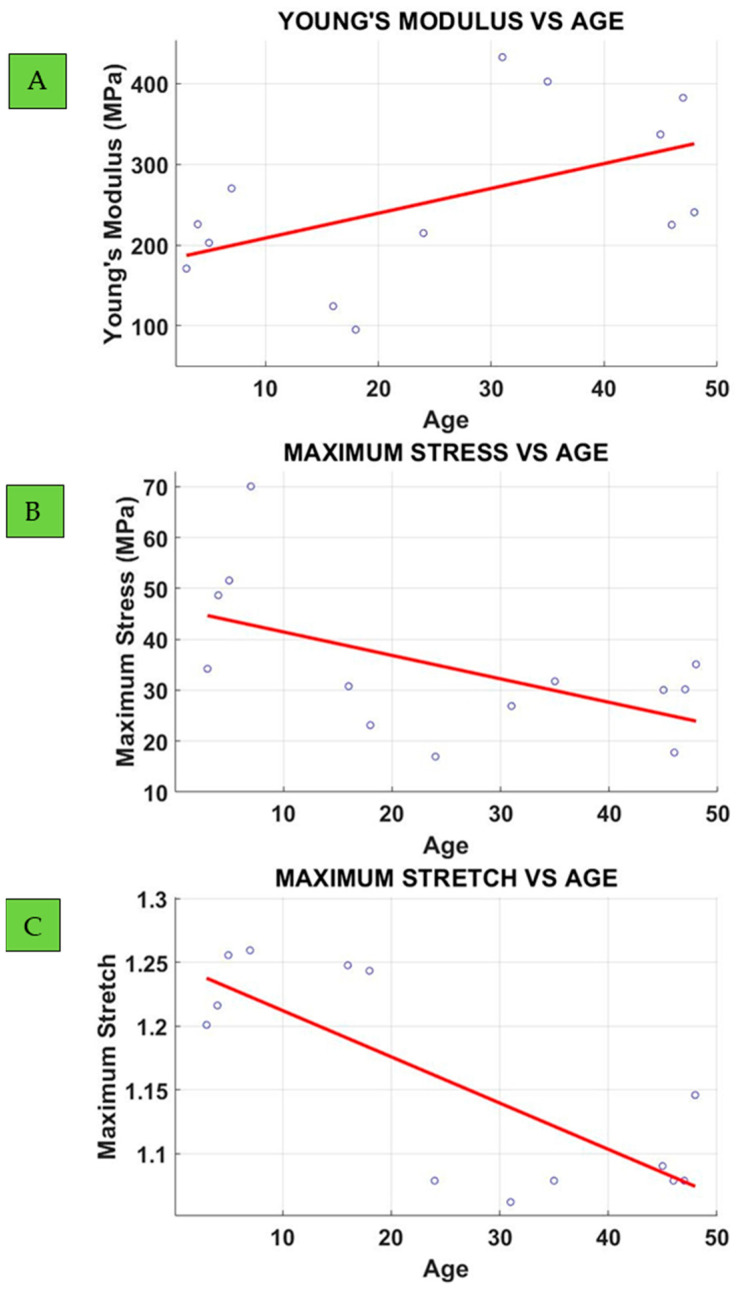
Effect of age on Young’s modulus (**A**), Maximum Stress (**B**) and Maximum Stretch (**C**).

The effect of age on Young’s modulus, maximum stress, and Maximum Stretch are shown in Figure 9.

The results indicate that for Young’s modulus, the slope is positive with a marginally significant *p*-value (0.0512) and a significant intercept (*p* = 0.0016), suggesting that the ligament loses compliance with age, with a reduction in Young’s modulus of approximately 3 MPa/year (Table 1), implying an increase of a 20% over the live span of the animal. In contrast, maximum stress and maximum stretch show a negative correlation with age. Maximum stress decreases by 0.46 MPa/year (*p* = 0.0346), as indicated in Table 1, implying a reduction of about 45% in the load-bearing capacity of the ligament over the lifespan of the animal. A similar trend is observed for maximum stretch, which decreases with age at a rate of 0.0036 1/year (*p* = 0.0007), implying a reduction of about 25% in maximum stretch over the animal’s lifespan.

Table 1 presents the results of the LMM analysis, summarizing the *p*-values for both the slope and intercept across the three mechanical properties: Young’s modulus, Maximum Stress and Maximum Stretch.

## 4. Discussion

We performed a tensile test on thirteen specimens of LCL at a moderately high strain rate. The systematic approach used allowed for the accurate characterization of the mechanical behaviour of the LCL across different ages, which is crucial for understanding how age-related factors may influence the biomechanical properties of these ligaments. The resulting stress–stretch curves provide valuable insights into the tensile properties, including maximum stress, Young’s modulus, and maximum stretch at failure. The experimental results reveal a significant decrease in maximum stress with age of the porcine LCLs. This aligns well with the study of Neumann [24], involving 15 human lumbar anterior longitudinal ligaments sourced from individuals spanning an age range of 30 to 80 years were studied. In this study, significant age-related declines in the ultimate load and ultimate stress of the human lumbar anterior longitudinal ligament are reported. The study also reveals a significant decrease in the maximum stretch, the reduction in bone mineral content, changes in collagen fibre composition, and decreased energy to failure with age collectively contribute to a lower maximum stretch capacity in older individuals [25].

Despite not being statistically significant, with the *p*-value very close to the significant limit of 0.05, the results show an increase in Young’s modulus with age in line with other studies in the literature. Changes in Young’s modulus can be associated with the loss of waviness of collagen fibres in ligaments with age [26], indicating that waviness plays a fundamental role in influencing this property. Older ligaments exhibit a greater number of fragmented and degenerated elastic fibres within their dense connective tissue, leading to a loss of normal compliance [27]. These alterations are closely associated with the deposition of large collagen fibres that break apart and envelop the elastic fibres. Additionally, ageing results in the disappearance of oxytalan fibres, which play a crucial role in providing tissue resistance [27]. Similar changes were noted in the skin [28] contributing to cutaneous wrinkling. Additionally, these changes were observed in the transversal fascia [29], promoting the development of inguinal hernias. In skeletal muscle [30,31], such alterations can lead to a reduction in force generation capacity, and in the splenic capsule, they can result in higher stiffness. These shared patterns of structural and quantitative changes across different tissues highlight the impact of ageing on tissue integrity and function.

Comparing our results to a study conducted in [32], which focused on the three samples of the lateral collateral ligament (LCL) in bone–ligament–bone (BLB) tests using 4–5 months old pigs, our stress values are notably higher. The reported LCL failure values near the tibia–ligament attachment were 6.225 MPa for stress and 1.16 for stretch. In our case, if we consider younger samples of the same average age, we obtained a maximum stress of 41.43 MPa and a stretch of 1.20. We observed a 6.5-times higher value of YM compared to the results [32]. This difference could be attributed to the higher strain rate in our study (13.3/s) compared to 0.02/s in the referenced study, as well as due to DB samples. In this study, the ligaments were preconditioned with 10 cyclic loading between 1 and 10 N at 0.167 mm/s while in our study, we loaded them from 0 to 20 N at 100 mm/s. In this study, a quick-setting bone cement was used while in our case, we solely rely on clamp force to hold the ligament. Furthermore, in a study by Bonner in 2014 [11], the mechanical properties of tissues from female large-white pigs aged between 9 and 12 months were investigated. Seven samples were tested at a strain rate of 10.06/s, which is reasonably comparable to our strain rate of 13.3/s. Bonner’s study, conducted in 2014 nearly a decade ago, reported maximum stress values approximately twice as high and Young’s modulus values nearly three times greater than those observed in the present study. Table 2 presents a comparative analysis of the results from Bonner versus our study. The standard deviation is provided in parentheses alongside the data point. Given that porcine tissues typically reach full maturity around 6–7 months of age, it is plausible that the reduction in maximum stress observed in our specimens is attributable to aging, as Bonner’s study utilized younger specimens (mean age: 10.5 months) compared to the older specimens in our study (mean age: 25.3 months). However, the notable differences observed with the values reported by Bonner et al. may reflect the impact of the methodological approach adopted for the mechanical testing as they used polymethyl-methacrylate (PMMA) for holding the bone, a drop impact tensile test instead of a uniaxial test, dog-bone versus bone–ligament–bone configurations, and inter-breed variability among porcine models. Furthermore, the limited availability of recent comparative data underscores the necessity for further investigations to contextualize these findings. These cumulative findings contribute valuable insights into the age-related biomechanical properties of pig ligaments and enhance our understanding of ligament behaviour under varying conditions.

When compared with results for the human LCL, the study by Smeets et al. [33] on 11 dog-bone specimens reported an average Young’s modulus of 289 ± 159.7 MPa, a maximum stress of 83.6 ± 38.1 MPa, and a maximum strain of 41.0 ± 9.9%. While the Young’s modulus was found to be similar to the value observed in this research, the maximum stress and maximum strain properties for the human LCL were higher, particularly in terms of maximum stretch, than those reported in our study. However, Smeets et al. did not provide information on the age of the specimens.

This study shows that as ligaments age, they become stiffer, less flexible, and less able to handle stress, which increases the risk of injury. Younger ligaments are better at adapting to mechanical loads, but over time, their ability to stretch and bear loads declines significantly. In medical practice, these insights can shape age-specific rehab programs to strengthen ligaments and prevent injuries. For surgeries, understanding how ligaments change with age can help surgeons choose the right grafts and set them up to match the patient’s needs. It also opens doors for treatments like tailored biomaterials or regenerative therapies to restore ligament function and slow down degeneration, giving patients better outcomes as they age. A recent study employed in vitro mechanical loading tests and numerical simulations to validate a model of osteophyte development, showing the impact of mechanical stress on chondrocyte viability and tissue degeneration [34]. While another recent study explores the application of mechanical principles in medical treatments, focusing on bone mechanobiology and cancerogenesis. It emphasizes the use of computational models and in silico trials to develop personalized therapies [35].

This method presents several limitations and challenges that impact the accuracy and reliability of the results. Notably, the physiological conditions of the porcine knee were not entirely replicated, despite maintaining constant hydration of the samples. The uneven surface presented a challenge during the sample preparation process. To address this, we focused on thoroughly cleaning the ligament to minimize its effects, although some residual challenges may still persist. Additionally, the DIC system we have is specifically designed for planar surfaces and is not suitable for 3D geometries. Consequently, we transitioned to using Tracker, a free and open-source software, for our data analysis. In some instances, slight slippage of the ligaments from the clamps’ grip was noted which may have introduced experimental bias. Furthermore, porcine specimens were sourced from different farms and varied in weight and size, which may have contributed to variability in the outcomes. Including male pigs and different breeds would enhance the reliability and generalizability of the results. However, due to logistical challenges, these factors were not addressed in the current study. A larger dataset, particularly including older porcine specimens, is required to draw definitive conclusions about the decline of the three markers, as they appeared relatively stagnant in terms of Young’s modulus and maximum stress between 18 and 48 months.

## 5. Conclusions

This study highlights the mechanical behaviour of the Lateral Collateral Ligament (LCL) and its dependence on age. Uniaxial mechanical testing on porcine samples aged 3 months to 4 years revealed significant age-related changes in the ligament’s properties. The Young’s modulus demonstrated a marginally significant positive trend, suggesting an increase in stiffness with age, while Maximum Stress and Maximum Stretch exhibited significant negative slopes, indicating a decline in the ligament’s strength and extensibility over time. These findings provide valuable insights into age-related biomechanical adaptations of the LCL, which may inform clinical approaches to injury prevention and treatment.

## Figures and Tables

**Table 1:** LMM analysis results.

Property	Slope (*p*-Value)	Intercept (*p*-Value)
Youngs modulus (MPa)	3.0753 (0.0512)	177.85 (0.0016)
Maximum Stress (MPa)	−0.4609 (0.0346)	46.052 (<0.0001)
Maximum Stretch (-)	−0.0036 (0.0007)	1.2484 (<0.0001)

**Table 2:** Result comparison Bonner et al., 2014 vs. our study.

Parameters	Bonner et al., 2014	Our Study
Max stress (MPa)	75.9 (9.6)	34.38 (14.12)
Max stretch	1.11 (0.03)	1.15 (0.078)
YM (MPa)	763 (141.3)	255.67 (100.94)
Strain rate (/s)	10.06	13.3
No of samples	7	13
Average age (months)	10.5 (0.87)	25.3 (17.02)

## Data Availability

Available upon request.

## References

[1] Akhtar R., Derby B., Derby B., Akhtar R. (2015). Introduction: Aging and the Mechanical Properties of Tissues. Mechanical Properties of Aging Soft Tissues.

[2] Woo S.L.Y., Ohland K.J., Weiss J.A. (1990). Aging and Sex-Related Changes in the Biomechanical Properties of the Rabbit Medial Collateral Ligament. Mech. Ageing Dev..

[3] van Gulick L., Saby C., Jaisson S., Okwieka A., Gillery P., Dervin E., Morjani H., Beljebbar A. (2022). An Integrated Approach to Investigate Age-Related Modifications of Morphological, Mechanical and Structural Properties of Type I Collagen. Acta Biomater..

[4] Davankar S.P., Deane N.J., Davies A.S., Firth E.G., Hodge H., Parry D.A.D. (2009). Collagen Fibril Diameter Distributions in Ligaments and Tendons of the Carpal Region of the Horse. Connect. Tissue Res..

[5] Parry D.A.D., Barnes G.R.G., Craig A.S. (1978). A Comparison of the Size Distribution of Collagen Fibrils in Connective Tissues as a Function of Age and a Possible Relation between Fibril Size Distribution and Mechanical Properties. Proc. R. Soc. Lond. B Biol. Sci..

[6] Lee T.Q., Dettling J., Sandusky M.D., McMahon P.J. (1999). Age Related Biomechanical Properties of the Glenoid-Anterior Band of the Inferior Glenohumeral Ligament-Humerus Complex. Clin. Biomech..

[7] Fremerey R., Bastian L., Siebert W.E. (2000). The Coracoacromial Ligament: Anatomical and Biomechanical Properties with Respect to Age and Rotator Cuff Disease. Knee Surg. Sports Traumatol. Arthrosc..

[8] The Strength of the Anterior Cruciate Ligament in Humans and Rhesus Monkeys. https://pubmed.ncbi.nlm.nih.gov/1002748/.

[9] Woo S.L.Y., Hollis J.M., Adams D.J., Lyon R.M., Takai S. (1991). Tensile Properties of the Human Femur-Anterior Cruciate Ligament-Tibia Complex. The Effects of Specimen Age and Orientation. Am. J. Sports Med..

[10] Cone S.G., Howe D., Fisher M.B. (2019). Size and Shape of the Human Anterior Cruciate Ligament and the Impact of Sex and Skeletal Growth: A Systematic Review. JBJS Rev..

[11] Bonner T.J., Newell N., Karunaratne A., Pullen A.D., Amis A.A., Bull A.M.J., Masouros S.D. (2015). Strain-Rate Sensitivity of the Lateral Collateral Ligament of the Knee. J. Mech. Behav. Biomed. Mater..

[12] He H., Deng Q., Wang C.X., Li J., Weng K.X., Miao Y.G. (2021). A Novel Methodology for Large Strain under Intermediate Strain Rate Loading. Polym. Test..

[13] Xerogeanes J.W., Fox R.J., Takeda Y., Kim H.S., Ishebashi Y., Carlin G.J., Woo S.L.Y. (1998). A Functional Comparison of Animal Anterior Cruciate Ligament Models to the Human Anterior Cruciate Ligament. Ann. Biomed. Eng..

[14] Farme Ihan—The Largest Pig Breeding Company in Slovenia. https://www.ihan.si/.

[15] Germscheid N.M., Thornton G.M., Hart D.A., Hildebrand K.A. (2011). A Biomechanical Assessment to Evaluate Breed Differences in Normal Porcine Medial Collateral Ligaments. J. Biomech..

[16] Cartner J.L., Hartsell Z.M., Ricci W.M., Tornetta P. (2011). Can We Trust Ex Vivo Mechanical Testing of Fresh--Frozen Cadaveric Specimens? The Effect of Postfreezing Delays. J. Orthop. Trauma..

[17] Woo S.L.Y., Orlando C.A., Camp J.F., Akeson W.H. (1986). Effects of Postmortem Storage by Freezing on Ligament Tensile Behavior. J. Biomech..

[18] Harris T., Coats T.J., Elwan M.H. (2018). Fluid Therapy in the Emergency Department: An Expert Practice Review. Emerg. Med. J..

[19] Miller T.E., Myles P.S. (2019). Perioperative Fluid Therapy for Major Surgery. Anesthesiology.

[20] Meyer J.P., McAvoy K.E., Jiang J. (2013). Rehydration Capacities and Rates for Various Porcine Tissues after Dehydration. PLoS ONE.

[21] STEP Lab—The Electrodynamic Testing Machine Experts. https://step-lab.com/.

[22] Digital Image Correlation (DIC) | 3D Full-Field Measurement. https://www.dantecdynamics.com/solutions/digital-image-correlation-dic/?gclid=Cj0KCQiAoae5BhCNARIsADVLzZc_a0TH5TZDyWA1dPcwf7bxJiEfcpP1-ACSZe8uOMwNK5XfLa_ypmkaAmxwEALw_wcB.

[23] Tracker Video Analysis and Modeling Tool for Physics Education. https://physlets.org/tracker/.

[24] Neumann P., Ekström L.A., Keller T.S., Perry L., Hansson T.H. (1994). Aging, Vertebral Density, and Disc Degeneration Alter the Tensile Stress-Strain Characteristics of the Human Anterior Longitudinal Ligament. J. Orthop. Res..

[25] Pintar F.A., Yoganandan N., Myers T., Elhagediab A., Sances A. (1992). Biomechanical Properties of Human Lumbar Spine Ligaments. J. Biomech..

[26] Maffulli N., Barrass V., Ewen S.W.B. (2000). Light Microscopic Histology of Achilles Tendon Ruptures: A Comparison with Unruptured Tendons. Am. J. Sports Med..

[27] Barros E.M.K.P., Rodrigues C.J., Rodrigues N.R., Oliveira R.P., Barros T.E.P., Rodrigues A.J. (2002). Aging of the Elastic and Collagen Fibers in the Human Cervical Interspinous Ligaments. Spine J..

[28] Iida T., Abumi K., Kotani Y., Kaneda K. (2002). Effects of Aging and Spinal Degeneration on Mechanical Properties of Lumbar Supraspinous and Interspinous Ligaments. Spine J..

[29] Quintas M.L., Rodrigues C.J., Yoo J.H., Rodrigues Junior A.J. (2000). Age Related Changes in the Elastic Fiber System of the Interfoveolar Ligament. Rev. Hosp. Clin. Fac. Med. Sao Paulo.

[30] .Rodrigues C.J., Rodrigues A.J. (2000). A Comparative Study of Aging of the Elastic Fiber System of the Diaphragm and the Rectus Abdominis Muscles in Rats. Braz. J. Med. Biol. Res..

[31] Rodrigues C.J., Rodrigues A.J., Bohm G.M. (1996). Effects of Aging on Muscle Fibers and Collagen Content of the Diaphragm: A Comparison with the Rectus Abdominis Muscle. Gerontology.

[32] Repositório Aberto Da Universidade Do Porto: Biomechanical Characterisation of Knee Ligaments: New Approach for Mechanical Testing and Computer Modelling. https://repositorio-aberto.up.pt/handle/10216/88097.

[33] Smeets K., Slane J., Scheys L., Claes S., Bellemans J. (2017). Mechanical Analysis of Extra-Articular Knee Ligaments. Part One: Native Knee Ligaments. Knee.

[34] Bednarczyk E., Sikora S., Jankowski K., Żołek-Tryznowska Z., Murawski T., Bańczerowski J., Lu Y., Senderowski C. (2024). Mathematical Model of Osteophyte Development with the First Attempt to Identify a Biomechanical Parameter. Contin. Mech. Thermodyn..

[35] Allena R., Rémond Y. (2023). Theramechanics: How acting on mechanics will help conceive new medical treatments. Math. Mech. Complex. Syst..

